# Extracellular signal-regulated kinase (ERK) pathway control of CD8^+^ T cell differentiation

**DOI:** 10.1042/BCJ20200661

**Published:** 2021-01-13

**Authors:** Marcos P. Damasio, Julia M. Marchingo, Laura Spinelli, Jens L. Hukelmann, Doreen A. Cantrell, Andrew J.M. Howden

**Affiliations:** 1 Division of Cell Signalling and Immunology, School of Life Sciences, University of Dundee, DD1 5EH Dundee, U.K.; 2 Centre for Gene Regulation and Expression, School of Life Sciences, University of Dundee, DD1 5EH Dundee, U.K.

**Keywords:** extracellular signal-regulated kinases, proteomics, T-cells

## Abstract

The integration of multiple signalling pathways that co-ordinate T cell metabolism and transcriptional reprogramming is required to drive T cell differentiation and proliferation. One key T cell signalling module is mediated by extracellular signal-regulated kinases (ERKs) which are activated in response to antigen receptor engagement. The activity of ERKs is often used to report antigen receptor occupancy but the full details of how ERKs control T cell activation is not understood. Accordingly, we have used mass spectrometry to explore how ERK signalling pathways control antigen receptor driven proteome restructuring in CD8^+^ T cells to gain insights about the biological processes controlled by ERKs in primary lymphocytes. Quantitative analysis of >8000 proteins identified 900 ERK regulated proteins in activated CD8^+^ T cells. The data identify both positive and negative regulatory roles for ERKs during T cell activation and reveal that ERK signalling primarily controls the repertoire of transcription factors, cytokines and cytokine receptors expressed by activated T cells. It was striking that a large proportion of the proteome restructuring that is driven by triggering of the T cell antigen receptor is not dependent on ERK activation. However, the selective targets of the ERK signalling module include the critical effector molecules and the cytokines that allow T cell communication with other immune cells to mediate adaptive immune responses.

## Introduction

The growth, proliferative expansion and differentiation of T lymphocytes is initiated by signalling pathways regulated by the T cell antigen receptor (TCR) and then balanced by positive and negative feedback signals transduced by cytokines, co-stimulatory receptors and inhibitory/immune checkpoint receptors [1]. The initial events in TCR signalling are mediated by cytosolic tyrosine kinases and adaptors that function to couple the TCR to a network of serine–threonine kinases that propagate the signal from the cell membrane to the nucleus and drive the transcriptional and metabolic changes that support effector T cell differentiation [2]. One important serine–threonine kinase signalling cascade regulated by the TCR is mediated by the mitogen-activated protein kinases (MAPK) ERK1 and ERK2 [3–5]. The activation of ERK1/2 in T cells is controlled by Ras guanine nucleotide-binding proteins. TCR triggering rapidly causes Ras proteins to cycle from a GDP-bound (inactive) to a GTP-bound (active) state that allows Ras proteins to bind to Raf serine–threonine kinases. This drives a pathway whereby active Raf kinases phosphorylate and activate the kinases MEK1/2 which then phosphorylate key threonine and tyrosine residues in ERK1/2 to activate these kinases [5,6].

The TCR acts as a digital switch for ERK1/2 activation in that the strength of the antigen stimulus determines the frequency of T cells that activate ERKs. This is a very sensitive switch with a low threshold for activation, and even very low levels of antigen receptor occupancy can activate the entire ERK1/2 pool in a T cell [4,7]. Moreover, ERK1/2 signalling is important for TCR function during positive selection in the thymus and in peripheral T cells. For example, activation of ERK2 is important for TCR induced T cell proliferation and differentiation and controls the survival of TCR activated peripheral CD8^+^ T cells [8]. There is also evidence that when ERK signalling pathways are inhibited during an *in vivo* immune response it prevents the differentiation of terminal effector cells and rather promotes the differentiation of memory ‘like’ T cells [9]. The importance of ERK1/2 activity for T cells and the sensitivity of flow cytometric assays to quantify the phosphorylation and hence the activation of ERK1/2 has promoted the use of this pathway as a sensitive readout of TCR receptor occupancy. However, although ERK1/2 activity is used to assess TCR signalling capacity the full details of how the RAS/MAPK cascade controls T cell function is not clear. Substrates for ERK1 and ERK2, that give some insights as to why this kinase pathway is so important in T cells, include the ternary complex factor subfamily of ETS-domain transcription factors ELK-1, SAP-1 and SAP-2, which control the expression of immediate early genes such c-Fos, Egr1 and Egr3 in T cells [10]. The ERK-mediated regulation of c-Fos expression contributes to the activator protein-1 (AP-1) transcription complex formed by the transcription factors c-Fos and Jun [11,12]. Egr1 and Egr3 play a critical role in T cell activation, regulating the expression of interleukin 2 (IL-2) and T cell proliferation [13,14]. There are also other ways in which ERK1/2 signalling can control T cell function. For example, the RAS/MAPK pathway can control microtubule remodelling through the phosphorylation and regulation of stathmin [15]. Moreover, many serine threonine kinases are phosphorylated and activated by the ERKs including the 90-kilodalton ribosomal protein S6 kinases (RSK1 and RSK2) which have been implicated in the control of cell cycle progression and cytokine production in activated T cells [16].

Quantitative analysis of T cell proteomes using high-resolution mass spectrometry is increasingly being used to understand T cell biology and provide new insight into how T cells respond to immune challenges and differentiate to effector cells [17–21]. The importance of such proteome analysis stems from the impact that changes in the rates of protein production (i.e. rates of protein synthesis versus degradation) can have on how a cell executes its transcriptional program. We have shown recently that antigen receptor engagement causes a remodelling of T cell proteomes by increasing and decreasing expression of more than 6000 and 1000 proteins, respectively [17]. Accordingly, the challenge is to understand the regulatory contribution of the different TCR signalling pathways to this proteome remodelling. In this context, we have recently mapped how the metabolic regulators mTORC1 and MYC control the proteome restructuring of immune activated cells [17,19]. The objective of the present report was to explore the ERK1/2 contribution as a control switch for antigen receptor-induced proteome remodelling. These data reveal that the ERK1/2 signalling pathway regulates a relatively small fraction of the proteome restructuring program initiated by the TCR. The data also identify both positive and negative regulatory roles for ERK1/2 signalling during T cell activation and show moreover that the dominant functions of ERK1/2 in T cells are to control the repertoire of transcription factors, cytokines and cytokine receptors expressed by activated T cells. The ERK1/2 controlled T cell proteins, while relatively small in number, includes many of the key molecules known to be pivotal for T cell differentiation and acquisition of effector function.

## Materials and methods

### Mice

For CD8^+^ TCR activated proteomes P14 [22] transgenic mice were used (female mice, 8–10 weeks old). Naïve CD8^+^ cells used for proteomics were described previously [19]. For the analysis of cell markers by flow cytometry P14 female mice aged 7 weeks were used. For cell proliferation assays male P14 mice aged 9–10 weeks were used, and for western blot analysis female P14 mice aged 14 weeks were used. Mice were euthanised by a schedule 1 method: asphyxiation by CO_2_ inhalation with confirmation of death by cervical dislocation. All mice were maintained in the Biological Resource Unit at the University of Dundee using procedures approved by the University Ethical Review Committee and under the authorisation of the U.K. Home Office Animals (Scientific Procedures) Act 1986.

### Cells and flow cytometry analysis

All cells were cultured at 37°C with 5% CO_2_ in RPMI 1640 containing glutamine (Invitrogen) and supplemented with 10% FBS (Gibco), 50 μM β-mercaptoethanol (Sigma) and penicillin/streptomycin (Gibco). For proteomics experiments *in vitro* TCR stimulation of lymphocytes was performed as follows: lymph nodes from P14 transgenic mice were removed and mashed in RPMI media in a 70 μm cell strainer. Cells were suspended in RPMI media and stimulated for 24 h with 100 ng/ml antigenic peptide GP33 (glycoprotein amino acids 33–41) and +/− 2 μM PD184352. After 24 h, cells were collected and prepared for cell sorting to generate a pure population of CD8^+^ T cells. Harvested cells were treated with 1 μg Fc block (BD Pharmingen) per million cells, to block Fc receptors. Cells were stained with CD8 PE and DAPI and sorted on an Influx cell sorter (Becton Dickinson). CD8^+^ viable cells were collected and washed with HBSS before being snap frozen in liquid nitrogen. Pure populations of naïve CD8^+^ T cells were prepared as described previously [19].

For flow cytometry experiments to examine surface marker and effector molecule expression, lymph nodes were removed from P14 mice and mashed and the cell suspension activated using the same conditions as for proteomics +/− inhibitor PD184352 (2 µM). Cells were placed in culture (37°C, 5% CO_2_) for 24 h. After activation cells were harvested by centrifugation. For staining cell surface markers, cells were blocked for 10 min with Fc block. Cells were stained with CD8 APC, CD69 PE, CD25 FITC, CD44 APC e780, all at 1 : 200 dilution in FACS buffer (DPBS 1% FBS) for 20 min at 4°C in the dark. Cells were washed and resuspended in FACS buffer before being analysed on a Novocyte flow cytometer. For intracellular staining of Granzyme B, cells were first washed with DPBS and labelled with live/dead fixable Aqua stain (Thermo Fisher Scientific) in DPBS for 15 min. Cells were then washed in FACS buffer, Fc blocked and stained with the surface marker CD8 FITC as described above. Stained cells were fixed with IC Fixation buffer (Thermo Fisher Scientific) for 30 min at room temperature, and then washed twice with permeabilization buffer (eBioscience) and stained with GZMB APC at 1 : 200 dilution in permeabilization buffer for 60 min at 4°C in the dark. Cells were then washed again with permeabilization buffer and analysed on a Novocyte flow cytometer.

For the analysis of phosphorylated ERK, splenocytes from P14 mice were suspended in RPMI media and stimulated for 48 h with 100 ng/ml antigenic peptide GP33 (glycoprotein amino acids 33–41) and 20 ng/ml interleukin-2 (IL-2) and 2 ng/ml interleukin 12 (IL-12). Cells were then washed out of activation media and cultured for a further 5 days in RPMI supplemented with 20 ng/ml IL-2 (media and IL-2 refreshed daily), to differentiate cytotoxic T cells (CTL). CTL were TCR stimulated for 4 h with GP33 peptide (100 ng/ml) +/− PD184352 (2 µM) or +/− IC87114 (PI3 kinase delta isoform inhibitor) at 10 µM. Cells were harvested by centrifugation and snap frozen.

For cell proliferation assays splenocytes freshly isolated from P14 mice were labelled with 5 μM CFSE (Invitrogen) at 37°C for 20 min in RPMI without FBS. Cells were labelled at a density of 10 × 10^6 ^cells/ml. Excess CFSE was washed off with RPMI with 10% FBS (complete media described above) and CFSE-labelled cells were seeded at 1 × 10^6 ^cell/ml and activated +/− 2 μM PD184352 with 100 ng/ml antigenic peptide GP33-41 in RPMI complete media. Unlabelled cells and CFSE-labelled cells incubated in media supplemented with IL-7 were used as controls. The proliferation of CD8^+^ T cells was assessed after 48 h by flow cytometric analysis by monitoring the dilution of CFSE label.

### Western blotting

Frozen pellets were lysed in RIPA buffer (100 mM HEPES, pH 7.4, 150 mM NaCl, 1% NP40, 0.1% SDS, 0.5% sodium deoxycholate, 10% glycerol, 1 mM EDTA, 1 mM EGTA, 1 mM TCEP, and protease and phosphatase inhibitors (Roche)). Cell lysates were sonicated and then centrifuged at 4°C at 16 000 g for 10 min. LDS sample buffer (Life Technologies) and tris(2-carboxyethyl)phosphine (TCEP), was added to samples at a final concentration of 1× and 25 mM, respectively, and samples were boiled for 10 min. Samples were loaded on a SDS–PAGE gel (NuPAGE precast gels, Life Technologies) and were then transferred to nitrocellulose membranes (Whatman). Blots were probed with the following antibodies: SMC1 (Bethyl Laboratories), phospho-ERK1/2 (p-p44/42 MAPK T202/Y204, Cell Signalling and Technology) and pan-ERK1/2 (p44/42 MAPK, Cell Signalling and Technology), followed by anti-mouse horseradish peroxidase (HRP)-conjugated secondary antibody (Thermo Scientific). Chemiluminescence was measured using an Odyssey Fc Imaging System (Licor).

### Proteomics sample preparation and peptide fractionation

Cell pellets were lysed as described previously [17]. In brief, cells were lysed in 400 μl lysis buffer (4% sodium dodecyl sulfate, 50 mM triethylammonium bicarbonate (pH 8.5) and 10 mM tris(2-carboxyethyl)phosphine-hydrochloride). Lysates were boiled for 5 min and sonicated with a BioRuptor (15 cycles: 30 s on and 30 s off) before alkylation with 20 mM iodoacetamide for 1 h at 22°C in the dark. Cell lysates were subjected to the SP3 procedure for protein clean-up [23] before elution into digest buffer (0.1% sodium dodecyl sulfate, 50 mM triethylammonium bicarbonate (pH 8.5) and 1 mM CaCl_2_) and digested with LysC and Trypsin, each at a 1 : 50 (enzyme : protein) ratio. Peptide clean-up was performed as described in the SP3 protocol [23] and peptides were eluted in 2% DMSO for fractionation.

Peptides from 24 h antigen-activated cells were fractionated using high pH reverse-phase chromatography as described previously [17]. Samples were loaded onto a XbridgeTM BEH130 C18 column with 3.5 μm particles (Waters). Using a Dionex BioRS system, the samples were separated using a 25-min multistep gradient of solvents A (10 mM formate at pH 9 in 2% acetonitrile) and B (10 mM ammonium formate at pH 9 in 80% acetonitrile), at a flow rate of 0.3 ml min^−1^. Peptides were separated into 16 fractions. The fractions were subsequently dried, and the peptides were dissolved in 5% formic acid and analysed by liquid chromatography-mass spectrometry. Peptides from naïve cells were fractionated as above but with slight modifications [19].

### Liquid chromatography-mass spectrometry analysis (LC-MS/MS)

LC–MS/MS was performed as described previously but with slight modifications [24]. For each sample, 1 μg of peptides was injected onto a nanoscale C18 reverse-phase chromatography system (UltiMate 3000 RSLC nano, Thermo Scientific) and electrosprayed into an Orbitrap mass spectrometer (LTQ Orbitrap Velos Pro; Thermo Scientific). For chromatography, the following buffer conditions were used: HPLC buffer A (0.1% formic acid), HPLC buffer B (80% acetonitrile and 0.08% formic acid) and HPLC buffer C (0.1% formic acid). Peptides were loaded onto an Acclaim PepMap100 nanoViper C18 trap column (100 µm inner diameter, 2 cm; Thermo Scientific) in HPLC buffer C with a constant flow of 10 µl/min. After trap enrichment, peptides were eluted onto an EASY-Spray PepMap RSLC nanoViper, C18, 2 µm, 100 Å column (75 µm, 50 cm; Thermo Scientific) using the buffer gradient: 2% B (0–6 min), 2–35% B (6–130 min), 35–98% B (130–132 min), 98% B (132–152 min), 98–2% B (152–153 min), and equilibrated in 2% B (153–170 min) at a flow rate of 0.3 µl/min. The eluting peptide solution was automatically electrosprayed using an EASY-Spray nanoelectrospray ion source at 50°C and a source voltage of 1.9 kV (Thermo Scientific) into the Orbitrap mass spectrometer (LTQ Orbitrap Velos Pro; Thermo Scientific). The mass spectrometer was operated in positive ion mode. Full-scan MS survey spectra (mass/charge ratio, 335–1800) in profile mode were acquired in the Orbitrap with a resolution of 60 000. Data were collected using data-dependent acquisition: the 15 most intense peptide ions from the preview scan in the Orbitrap were fragmented by collision-induced dissociation (normalised collision energy, 35%; activation Q, 0.250; activation time, 10 ms) in the LTQ after the accumulation of 5000 ions. Precursor ion charge state screening was enabled, and all unassigned charge states as well as singly charged species were rejected. The lock mass option was enabled for survey scans to improve mass accuracy.

### Proteomics data processing and analysis

Raw mass spec data files were searched using the MaxQuant software package (version 1.6.2.6). Proteins and peptides were identified using a hybrid database from the Uniprot release 2019 07. The hybrid database was generated using all manually annotated mouse SwissProt entries, combined with mouse TrEMBL entries with protein level evidence available and a manually annotated homologue within the human SwissProt database. The following search parameters within MaxQuant were selected: protein N-terminal acetylation and methionine oxidation were set as variable modifications and carbamidomethylation of cysteine residues was selected as a fixed modification; trypsin and LysC were selected as digestion enzymes with up to 2 missed cleavages; the false discovery rate was set at 1% for protein and peptide and the match between runs function was disabled. Proteins were removed from the data set which were categorised as ‘reverse’, ‘contaminant’ or ‘only identified by site’. Estimates of protein copy numbers per cell were calculated using the histone ruler method [25] within the Perseus software package [26].

### Statistics and calculations

To identify significant changes in protein abundance *P*-values were calculated using a two-tailed *t*-test with unequal variance, on log-normalised protein copy numbers. Proteins were considered to change significantly with a *P*-value < 0.05 and a fold change >1.5 or <0.66. To identify processes that may be enriched upon ERK1/2 inactivation a more stringent fold change cut-off was established. The standard deviation of the log2(fold change) for all proteins was calculated and a significance cut-off was set as 2 standard deviations from the mean log2(fold change). Gene ontology enrichment analysis was performed using the database for annotation, visualisation and integrated discovery (DAVID) v6.8 [27]. The mass of individual proteins was estimated using the following formula: CN × MW/NA = protein mass (g cell^−1^), where CN is the protein copy number, MW is the protein molecular mass (in Da) and NA is Avogadro's Constant. Heat maps were generated using the Morpheus tool from the Broad Institute (https://software.broadinstitute.org/morpheus). Only proteins with a copy number of at least 500 in all 3 replicates and at least 1 cell population (naïve, TCR activated or TCR activated + inhibitor) were included in the heat map.

## Results

### ERK1/2 selectively remodels the proteome of antigen receptor-activated T cells

**Figure 1.**
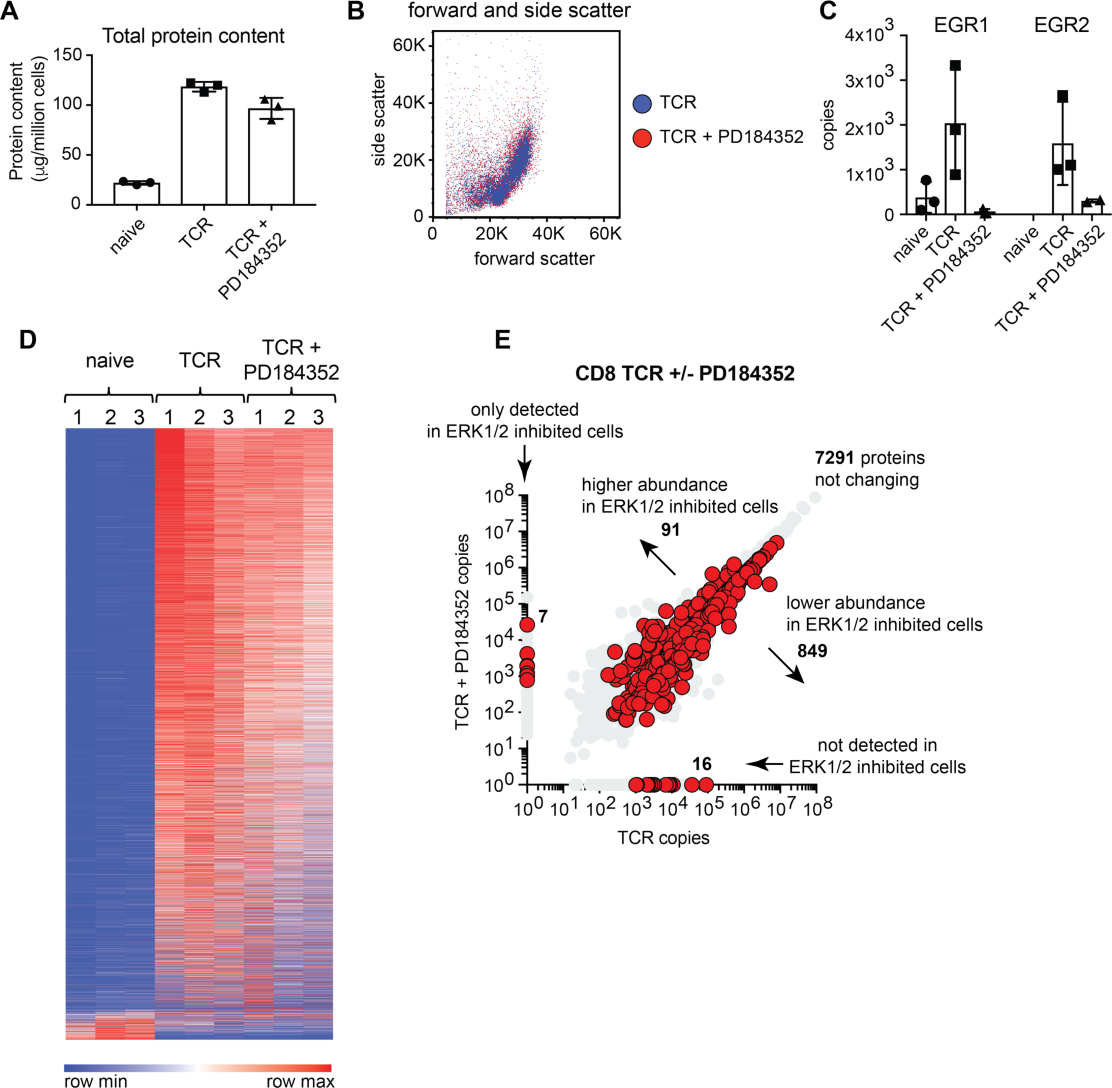
Selective proteome remodelling by ERK1/2. High-resolution quantitative mass spectrometry was used to characterise the proteomes of naïve and 24 h antigen-activated CD8^+^ T cells +/− PD184352, a selective inhibitor of ERK1/2 activation. (**a**) Total protein content of T cell populations. (**b**) Forward/side scatter flow cytometry analysis of control and inhibitor-treated cells after 24 h of antigen activation. (**c**) Mean protein copy numbers per cell of the transcription factors early growth response 1 and 2 (EGR1 and EGR2). (**d**) Heat map of naïve and TCR activated CD8^+^ T cell proteomes +/− PD184352. Abundance is graded from low (blue) to high (red) for each individual protein. (**e**) Protein copy number comparison for control and inhibitor-treated cells. Proteins highlighted in red were significantly different between the two populations (*P*-value < 0.05, fold change <0.66 or >1.5, 2-tailed *t*-test with unequal variance) or were exclusively found in one population at >500 copies per cell and were not detected in the other population. For proteomics data, copy numbers are the mean of 3 biological replicates +/− standard deviation.

PD184352 is a highly selective inhibitor of MEK [28], which leads to effective inhibition of TCR-induced ERK1/2 activity (Supplementary Figure S1). To explore how ERK signalling controls T cells, quantitative high-resolution mass spectrometry was used to resolve proteomes of CD8^+^ T cells after 24h of antigen activation in the presence or absence of PD184352. These experiments used P14 CD8^+^ T cells which express a TCR specific for lymphocytic choriomeningitis virus glycoprotein peptide GP33-41 [22] and hence allow an assessment of how ERK1/2 signalling shapes T cell responses to peptide/MHC complexes. We also compared the proteomes of activated cells to those of naïve CD8^+^ T cells, allowing evaluation of the impact of ERK1/2 activity on TCR induced proteome remodelling. We identified over 8000 proteins and estimated protein copy numbers per cell and protein abundance using the ‘proteomic ruler’ method which uses the histone mass spectrometry signal as an internal standard [25] (Supplementary File S1). Antigen activation of naïve CD8^+^ T cells caused a large increase in total cell protein content (Figure 1A). Interestingly, blocking ERK1/2 activity had only a modest effect on the mass of antigen-activated cells (Figure 1A), with cells still achieving a large increase in protein content compared with naïve cells. This was supported by flow cytometry data of cell forward and side scatter, which confirmed that blocking ERK1/2 activity had only a modest impact on the estimated size of these T cells after 24 h of TCR activation (Figure 1B). To assess the efficacy of the ERK1/2 inhibitory strategy we first looked at the effects of PD184352 on the expression of previously defined targets for ERK1/2 signalling pathways in T cells: the transcription factors EGR1 and EGR2. The data show that the up-regulation of EGR1 and 2 expression which normally occurs in antigen-activated T cells was suppressed when ERK1/2 activity was blocked (Figure 1C).

**Figure 2.**
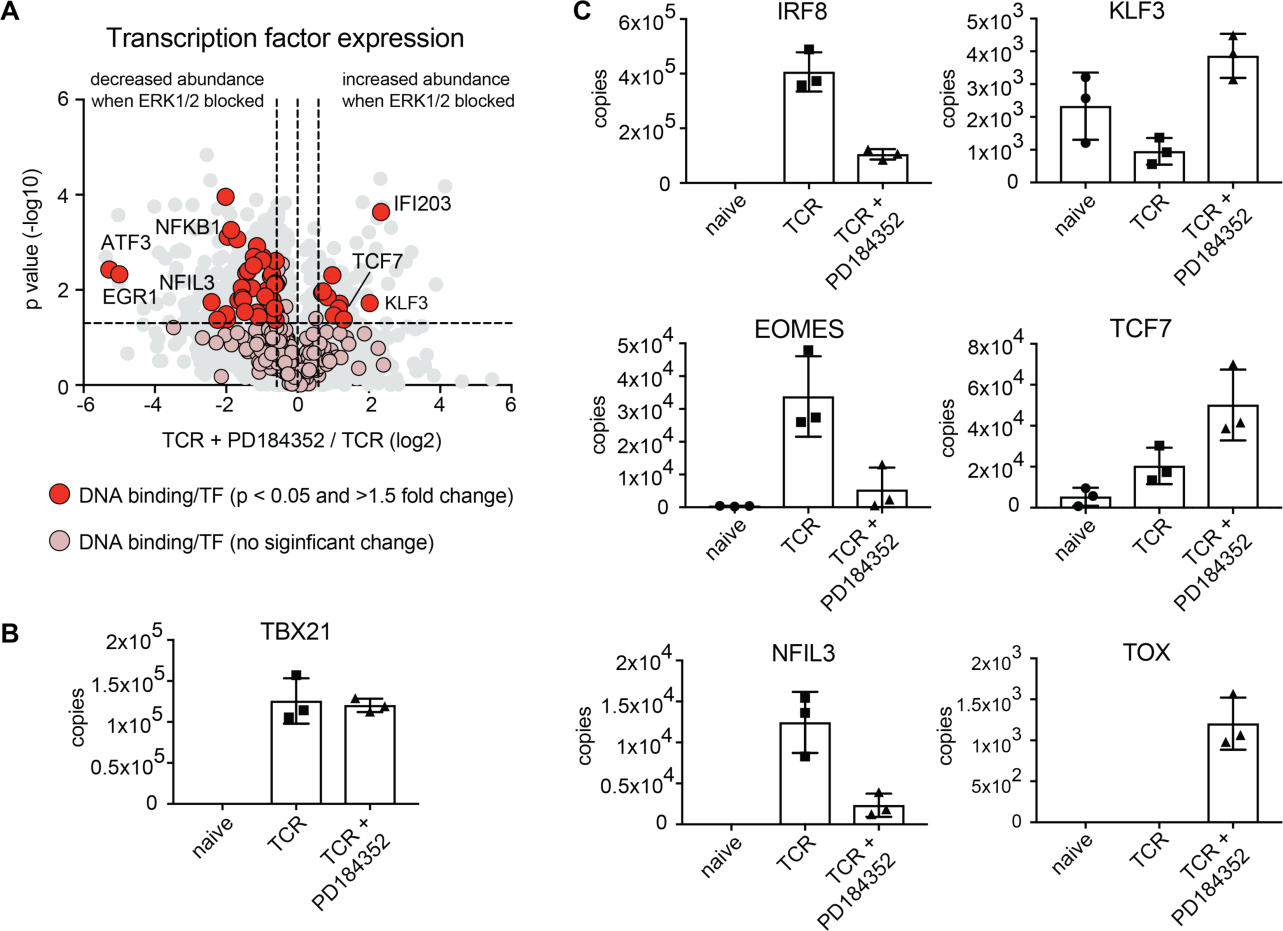
ERK1/2 activity is critical for the expression of key transcription factors during CD8^+^ T cell activation. (**a**) The expression profile of over 300 proteins with the gene ontology term GO:0003700 (DNA binding transcription factor activity) was assessed in response to blocking ERK1/2 activity (TCR + PD184352). Proteins highlighted in red were annotated with the above GO term and were significantly different between the two populations (*P*-value < 0.05, fold change <0.66 or >1.5, 2-tailed *t*-test with unequal variance). Proteins highlighted in pink are transcription factors that did not significantly change in response to blocking ERK activity. Mean protein copy number per cell of (**b**) TBX21, T-Box Transcription Factor 21 (T-bet), (**c**) IRF8, IFN regulatory factor 8; EOMES, eomesodermin; NFIL3, Nuclear Factor Interleukin 3 Regulated; KLF3, Kruppel Like Factor 3; TCF7, Transcription factor 7; TOX, Thymocyte Selection-Associated High Mobility Group Box. Copy numbers are the mean of 3 biological replicates +/− standard deviation.

We next evaluated the global protein expression profile of CD8^+^ T cells activated with antigen with and without ERK1/2 activity using nearest neighbour analysis and Pearson correlation (Figure 1D). We have previously shown that antigen activation triggers significant proteome remodelling in CD8^+^ T cells with thousands of proteins increasing in abundance while a smaller proportion of proteins drop in abundance in response to activation [17]. In the current study, we found that antigen-activated T cells still underwent a striking proteome remodelling despite ERK1/2 activity being blocked (Figure 1D and Supplementary Figure S2). Of the 8000 proteins quantified in activated T cells, over 7000 proteins did not change in abundance when ERK1/2 activity was blocked (Figure 1E). However, blocking ERK1/2 activity did decrease the expression of approximately 800 proteins while almost 100 proteins were found at higher levels when ERK1/2 activity was blocked (Figure 1E and Table 1). There were also examples of proteins whose expression was completely absent from one population versus the other (Figure 1E and Table 2). For example, lymphotoxin alpha (LTα), tumour necrosis factor (TNF) and IL-2, were detected in 24 h TCR activated control cells but not detected in ERK1/2 inhibited cells, while the cell exhaustion associated protein Thymocyte selection-associated high mobility group box protein TOX was not normally found in antigen receptor-activated T cells but was detected when T cells were antigen-activated but ERK activation was blocked (Table 2). Ten other transcription factors were also found at higher levels in inhibitor-treated cells versus control including Kruppel Like Factor 3 (KLF3) and transcription factor 7 (TCF7 or TCF-1) (Figure 2A–C). In this context, T cell receptor activation results in significant changes in the transcription factor profile of CD8^+^ T cells [17]. In this study over 300 proteins annotated as transcription factors were identified and the majority of these did not change significantly in response to ERK1/2 inactivation (Figure 2A and Supplementary File S1). For example, T-Box Transcription Factor 21 (T-BET/TBX21) is critical for T cell differentiation and was still significantly up-regulated in antigen-activated T cells when ERK1/2 activity was blocked (Figure 2B). However, inhibition of ERKs did cause reduced expression of many critical transcription factors that are normally increased in expression in activated CD8^+^ T cells including interferon regulatory factor 8 (IRF8), Eomesodermin (EOMES) and nuclear factor interleukin 3 regulated (NFIL3). These transcription factors all play key roles controlling effector T cell differentiation [17,29–32] and were all significantly reduced in abundance when ERK1/2 activity was blocked (Figure 2C).

**Table 1.** Number of proteins changing in abundance in response to blocking ERK1/2 activity

	Number of proteins
Lower abundance in ERK1/2 inhibited cells	849
Not detected in ERK1/2 inhibited cells	16
Higher abundance in ERK1/2 inhibited cells	91
Only detected in ERK1/2 inhibited cells	7
Not changing	7291

Proteins were considered to change significantly with a *P*-value <0.05 and a fold change <0.66 or >1.5 (2-tailed *t*-test with unequal variance). Proteins were classified as having a presence/absence expression profile if they were exclusively found at >500 copies per cell in one population but absent from the other population.

**Table 2.** Proteins showing a presence/absence expression profile in response to blocking ERK1/2 activity

Protein name	Gene name	naïve	TCR	TCR + PD184352
RNA-binding protein MEX3D	Mex3d		89 700	
Lymphotoxin alpha	Lta		36 100	
Tumor necrosis factor	Tnf		10 900	
T-complex protein 1 subunit zeta-2	Cct6b	1100	9100	
Plasminogen activator inhibitor 1	Serpine1		8400	
Cytokine-inducible SH2-containing protein	Gish		8200	
Runt-related transcription factor 2	Runx2		6900	
Unconventional myosin-X	Myo10		3300	
Protein BTG3	Btg3		3000	
Tumor necrosis factor ligand superfamily member 8	TnfsfB		2600	
HemK methyltransferase family member 1	Hemk1		2500	
Mitogen-activated protein kinase kinase kinase 8	Map3k8		2300	
lnterleukin-2	112		2200	
Solute carrier organic anion transporter family member 4A1	Slco4a1		2100	
Regulator of nonsense transcripts 1	Upf1		1100	
Psychosine receptor	Gpr65		1000	
Src-like-adapter 2	Sla2	500		26 500
Drebrin-like protein	Dbnl			4100
Spindle and kinetochore-associated protein 2	Ska2			1900
Peroxisomal membrane protein 4	Pxmp4	600		1900
Thymocyte selection-associated high mobility group box protein TOX	Tox			1200
Class II histocompatibility antigen, M alpha chain	H2-DMa			1000
BCL2-like 12	Bcl2I12			800

Proteins were considered to have a presence/absence expression profile if they were found in all 3 biological replicates of one condition at a copy number >500 copies per cell and were not detected in any biological replicates of the comparison condition.

### ERK1/2 activity controls the expression of effector molecules, cytokines and cytokine receptors in activated CD8^+^ T cells

**Figure 3.**
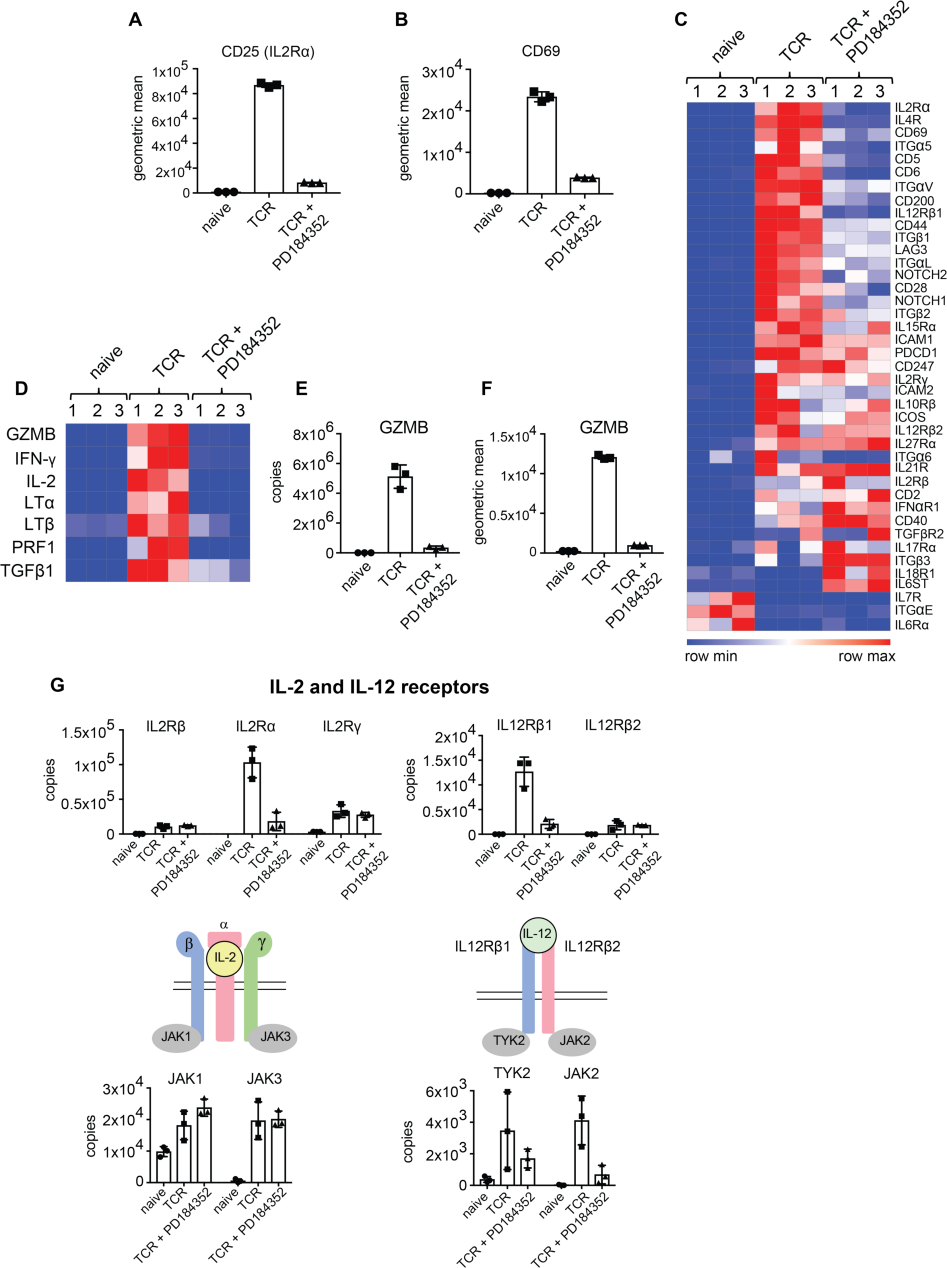
ERK1/2 activity controls the expression of effector molecules, cytokine receptors and their downstream signalling components. (**a**,**b**) Flow cytometry analysis of (**a**) CD25 (IL2Rα) and (**b**) CD69, in naïve and TCR activated CD8^+^ T cells +/− PD184352. Data show the geometric mean for 3 biological replicates. (**c**) Expression profile of cell surface receptors in naïve and TCR activated CD8^+^ T cells +/− PD184352. The heat map shows the relative abundance of individual proteins graded from low (blue) to high (red). (**d**) Expression profile of effector molecules in naïve and antigen-activated CD8^+^ T cells +/− PD184352. GZMB, granzyme B; IFN-γ, interferon-gamma; IL-2, interleukin 2; LTα, lymphotoxin alpha; LTβ, lymphotoxin beta; PRF1, perforin 1; TGFβ1, Transforming Growth Factor Beta 1. (**e**) Mean protein copy numbers per cell of GZMB. (**f**) Flow cytometry analysis of GZMB in naïve and TCR activated CD8^+^ T cells +/− PD184352. The graph shows the geometric mean for 3 biological replicates. (**g**) The abundance of IL-2 and IL-12 receptor subunit components. The IL-2 receptor consists of three subunits: IL-2 receptor subunit alpha, beta and gamma (IL2Rα, IL2Rβ and IL2Rγ), while the IL-12 receptor consists of two subunits: IL-12 receptor subunit beta 1 and 2 (IL12Rβ1 and IL12Rβ2). JAK, Janus kinase. TYK2, Tyrosine Kinase 2. For proteomics data, copy numbers are the mean of 3 biological replicates +/− standard deviation.

Flow cytometry analysis shows that blocking ERK1/2 activity impaired the ability of T cells to up-regulate expression of the IL-2 receptor alpha subunit (IL2Rα/CD25), and the adhesion molecule CD69 (Figure 3A,B). The expression of CD25 and CD69 is used frequently to monitor T cell activation and in this context, a gene ontology enrichment analysis for proteins that showed the greatest drop in abundance in T cells treated with PD184352 (Table 3) revealed that these included many other key membrane proteins found on activated T cells. The top enriched GO term applied to ERK1/2 regulated proteins was GO BP:0006955 or ‘immune response’ and cell surface proteins down-regulated in ERK1/2 inhibited T cells included CD5, CD6, CD200, CD69, LAG3 and the integrins ITGα5, ITGαL, ITGαV, ITGβ1 and ITGβ2 and the Interleukin 4 receptor (IL4R) (Figure 3C). The loss of ERK1/2 signalling was also associated with increased expression of the Interleukin 18 receptor I and the Interleukin 6 signal transducer (IL6ST), the signalling subunit of the IL-6 receptor (Figure 3C and Table 4). It is also relevant that the immune response GO BP:0006955 annotation also includes many of the key CD8^+^ T cell effector molecules and one very clear result was that ERK signalling was required for the normal expression of multiple effector molecules essential for CTL function (Table 3 and Figure 3D). ERK signalling pathways thus controlled expression of interferon-gamma (IFN-γ), IL-2, lymphotoxin alpha and beta (LTα and LTβ), transforming growth factor-beta 1 (TGFβ1), perforin (PRF1) and granzyme B (GZMB), (Figure 3D–F).

**Table 3.** Gene ontology enrichment analysis for those proteins that showed reduced abundance when ERK1/2 activity was blocked

Gene name	Protein name
Top enriched GO term — immune response GO:0006955	
lfn-γ	Interferon-gamma
II2rα	Interleukin 2 receptor, alpha chain
II2	Interleukin 2
Ltα	Lymphotoxin alpha
Nfil3	Nuclear factor, interleukin 3 regulated
Serpinb9	Serine (or cysteine) peptidase inhibitor, clade B, member 9
Tnfsf11	Tumor necrosis factor (ligand) superfamily, member 11
Tnfsf8	Tumor necrosis factor (ligand) superfamily, member 8
Tnfrsf18	Tumor necrosis factor receptor superfamily, member 18
Tnfrsf4	Tumor necrosis factor receptor superfamily, member 4
Tnf	Tumor necrosis factor

Proteins that showed the greatest drop in abundance (*P*-value < 0.05 and fold change >2 standard deviations from the mean fold change; two-tailed *t*-test with unequal variance) or a presence/absence expression profile, were subject to enrichment analysis for biological processes. The top enriched process is presented ‘immune response’ GO:0006955, along with a list of those proteins identified within this enrichment group.

**Table 4.** The expression profile of cytokine receptors in response to blocking ERK1/2 activity

	naïve	TCR	TCR + PD184352	Fold change	*P*-value
**IL2Rα**		103 000	18 000	0.2	0.03
IL2Rβ	200	10 400	12 200	1.2	0.35
IL2Rγ	3100	33 000	27 700	0.8	0.42
**IL4R**	60	3200	500	0.2	0.00
IL6Rα	700		200		
**IL6ST**	900	300	4700	17.6	0.00
IL7R	2000				
IL10Rβ		2500	1900	0.8	0.76
**IL12R**β**1**		12 700	2100	0.2	0.01
IL12Rβ2		1800	1800		0.78
IL15Rα		5400	3400	0.62	0.16
IL17Rα		400	500	1.3	0.79
IL18R1	800	200	3000	12.8	
IL21R	400	900	200	0.2	0.07
IL27Rα	300	1000	1000		

Mean protein copy numbers per cell for cytokine receptors identified within naïve and 24 h antigen-activated CD8^+^ T cells +/− PD184352. The fold change (TCR + inhibitor/TCR) is presented along with *P*-value (two-tailed *t*-test with unequal variance). Proteins highlighted in bold showed a significant change in expression when ERK activity was blocked (*P*-value < 0.05 and a fold change <0.66 or >1.5).

These data highlight that one critical function for ERK signalling in T cells is to control the expression of T cell membrane proteins that control the ability of T cells to respond to immune cues. In this context, two key cytokines for CD8^+^ T cell differentiation are IL-2 and IL-12 which signal through multi-subunit receptors that couple to the Janus family tyrosine kinases. The high-affinity IL-2 receptor comprises three subunits IL2Rα (CD25), IL2Rβ and IL2Rγ, whereas the IL-12 receptor is a dimer of IL12Rβ1 and IL12Rβ2. The flow cytometry data in Figure 3A shows that blocking ERK signalling severely blunted the normal up-regulation of the expression of CD25/IL2Rα. The proteomic data put some numbers to the magnitude of this effect in that CD25 was not detected in naive T cells, was expressed at 100 000 copies in TCR activated cells but only 18 000 copies when ERK1/2 activity was blocked (Table 4 and Figure 3G, see also the flow cytometry data in Figure 3A). The data also reveal that ERK signalling pathways did control the expression of the IL12Rβ1 subunit which was not detected in naive T cells, expressed at almost 13 000 copies in TCR activated cells but only 2000 copies when ERK1/2 activity was blocked (Table 4 and Figure 3G). Interestingly, there was no ERK requirement for antigen receptor-induced increases in the expression of IL2Rβ or IL2Rγ subunits or the IL12Rβ2 subunit (Figure 3G).

The IL-2 receptor signals via JAK1 which binds to IL2Rβ and JAK3 which binds to IL2Rγ. The IL-12 receptor signals through TYK2 and JAK2 which bind respectively to IL12Rβ1 and IL12Rβ2. One new insight from the proteomic data is that antigen receptor engagement controls the expression of the key tyrosine kinases needed for signal transduction by the IL-2 and IL-12 receptors. Naive T cells were found to constitutively express relatively high levels of JAK1 (approximately 10 000 copies per cell) but only very low levels of JAK2, JAK3 and TYK2 (approximately 50, 500 and 400 copies, respectively). The expression of JAK1 increased approximately 2-fold in immune activated T cells whereas increases in expression of JAK2, JAK3 and TYK2 were much more striking, increasing between 10 and 100-fold (Figure 3G). Interestingly, ERK signalling did not equally mediate antigen receptor control of the expression of the different JAKs. For example, ERK1/2 activation was required for antigen receptor up-regulation of TYK2 and JAK2 but not needed for the expression of JAK1 or JAK3. These results provide the insight that integration of multiple signalling pathways is needed for something as simple as the up-regulation of individual cytokine receptor complexes and associated signalling molecules. However, the salient point is that ERK signalling does control the expression of key subunits and signalling components of important cytokine receptors for CD8^+^ T cell differentiation.

### ERKs are not the dominant regulators of T cell metabolic and biosynthetic programs

**Figure 4.**
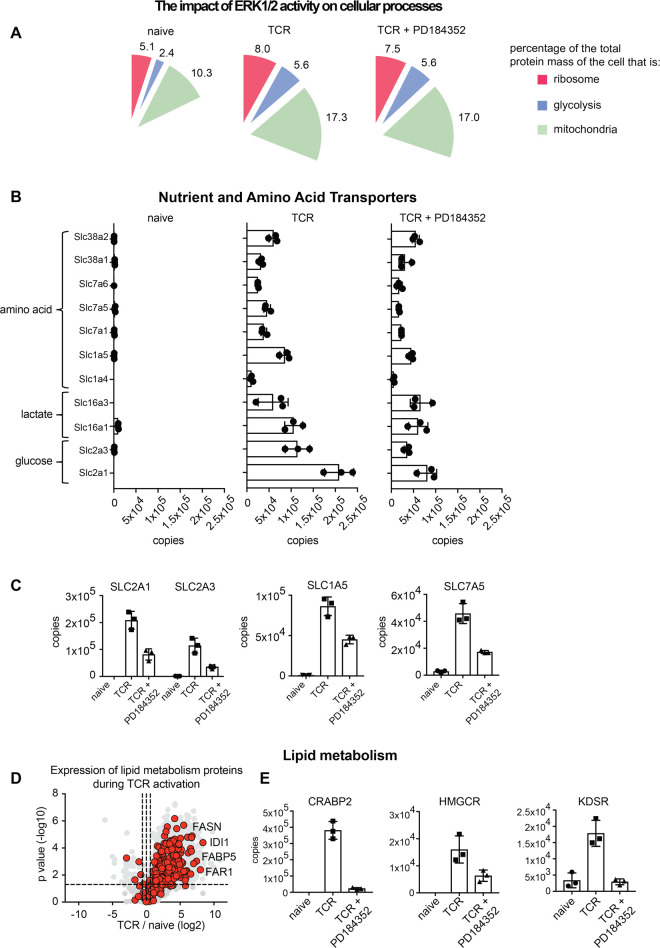
The impact of ERK activity on metabolic processes. (**a**) Percentage of the total cellular protein mass that represents ribosomal, glycolytic or mitochondrial proteins. (**b**,**c**) Expression profile of the major transporters for amino acids, lactate and glucose. (**d**) The impact of antigen activation on the expression of proteins linked to lipid metabolic processes (GO:0006629). Volcano plot shows the ratio for 24 h antigen-activated cells versus naïve cells. The horizontal dashed line indicates a *P*-value of 0.05 while the outer vertical dashed lines indicate a fold change of 0.66 and 1.5. IDI1, Isopentenyl-diphosphate Delta-isomerase 1; FAR1, Fatty acyl-CoA reductase 1; FABP5, Fatty acid-binding protein 5; FASN, Fatty acid synthase. (**e**) The mean copy number per cell for a selection of lipid metabolic proteins which were significantly impacted when ERK activity was blocked: CRABP2, Cellular Retinoic Acid-Binding Protein 2; HMGCR, 3-Hydroxy-3-Methylglutaryl-CoA Reductase; KDSR, 3-Ketodihydrosphingosine Reductase. Copy numbers are the mean of 3 biological replicates +/− standard deviation.

Regulated changes in T cell metabolism are essential for T cell function [33,34] so it was important to evaluate whether critical cellular metabolic compartments and processes are regulated by the ERK signalling pathway. Ribosomes, glycolytic enzymes and mitochondria make up a larger proportion of the mass of an activated T cell versus a naïve T cell: 5%, 2% and 10% in a naïve cell versus 8%, 5% and 17% in a TCR activated cell (Figure 4A). The increase in cellular protein mass devoted to these processes highlights the shift in the metabolic demand of T cells during activation. ERK1/2 activity was not required for this remodelling of the T cell proteome and T cells with ERK activity blocked still dramatically increased their ribosome, glycolysis and mitochondrial protein mass (Figure 4A). We also looked at how the inhibition of ERKs impacted the regulated expression of amino acid and glucose transporters that deliver the key nutrients that fuel T cell metabolic processes. In this context, we have shown recently that T cells that fail to up-regulate the System L amino acid transporter SLC7A5 remain small and have lower expression of >3000 proteins [19]. The data in Figure 4B show that in the absence of ERK activation, antigen receptor-activated T cells still increase expression of essential amino acid, glucose and lactate transporters. In the absence of ERK signalling the increases in SLC7A5, SLC1A5 (neutral amino acid transporter) and SLC2A1 and SLC2A3 (glucose transporters), were blunted and approximately 2-fold lower than the increases seen in control activated cells (Figure 4C). However, given that we saw no major impact of ERK inhibition on cell mass (Figure 1A) we infer that this modest decrease in expression of the nutrient transporters was not limiting for T cell growth.

We also explored the impact of ERK activity on lipid metabolism by examining the expression profiles of proteins that mediate lipid metabolic processes in naive and TCR activated T cells and in T cells activated but with ERK activity blocked (Figure 4D,E). We identified over 400 proteins which were annotated as being linked to lipid metabolic processes (GO:0006629). Lipid metabolism was found to be highly TCR regulated with most proteins increasing significantly when naïve CD8^+^ T cells were antigen-activated (Figure 4D). Indeed, some lipid metabolism proteins increased >100 fold upon TCR activation including fatty acyl-CoA reductase 1 (FAR1) which increased from 500 copies to 150 000 copies and isopentenyl-diphosphate delta-isomerase 1 (IDI1) which increased from 4000 copies to over 1.3 million copies when naïve CD8^+^ cells were TCR triggered (Figure 4D and Supplementary File S1). Blocking ERK activation impacted the expression of many lipid metabolism proteins including cellular retinoic acid-binding protein 2 (CRABP2), hydroxymethylglutaryl-CoA reductase (HMGCR) and 3-ketodihydrosphingosine reductase (KDSR) (Figure 4E). The drop in abundance of CRABP2 when ERK activation was blocked was especially striking, falling from almost 400 000 copies in control TCR activated cells to just over 20 000 copies in ERK inhibited cells. CRABP2 is a retinoic acid (RA) transporter which in turn controls the expression of RA target genes. CRABP2 and RA are key regulators of the differentiation and activity of immune cells [35]. Nevertheless, the key conclusion overall from these analyses is that ERKs are not the dominant regulators of T cell metabolic and biosynthetic programs.

### ERK1/2 control of CD8^+^ T cell proliferation and survival

**Figure 5.**
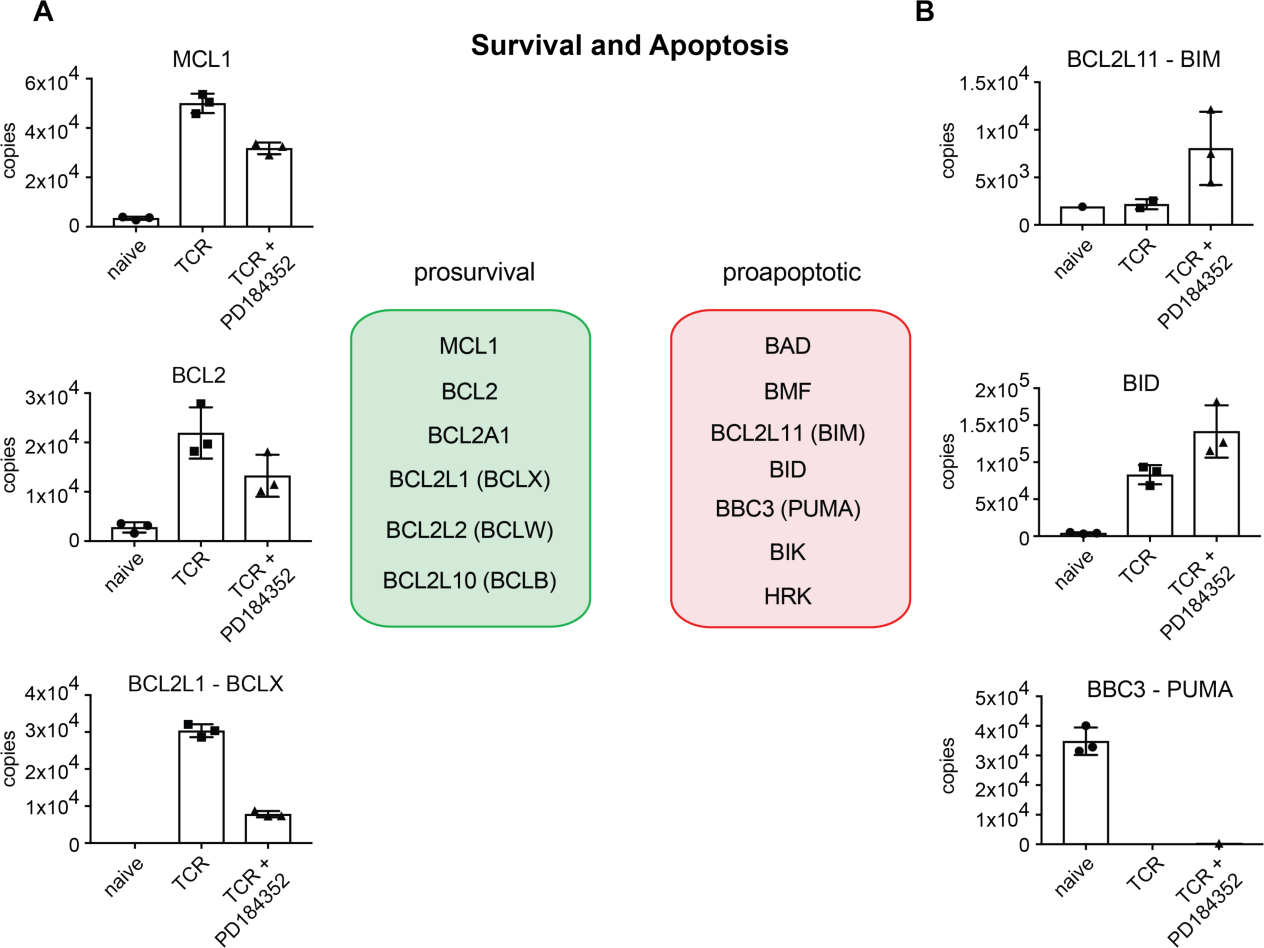
Blocking ERK activity directs cells towards an apoptotic profile. The expression profile of key pro-survival (**a**) and pro-apoptotic (**b**) proteins was assessed in response to blocking ERK activity. MCL1, MCL1 apoptosis regulator BCL2 family member; BCL2, BCL2 apoptosis regulator; BCL2L1 (BCLX), BCL2 Like 1; BCL2L11 (BIM), BCL2 like 11; BID, BH3 Interacting Domain Death Agonist; BBC3 (PUMA), BCL2 Binding Component 3. Copy numbers are the mean of 3 biological replicates +/− standard deviation.

The magnitude of the T cell proliferative response is determined by the rate of T cell cycle progression, the total number of cell divisions performed and the ability of T cells to survive during the proliferative expansion phase [36,37]. In this respect, previous studies have shown that the ERK signalling pathway is critical for T cell proliferative expansion and survival [8]. We, therefore, interrogated the proteomic data to understand how ERKs control T cell survival and proliferation. The data show that immune activation induced an increase in quantities of three pro-survival BCL2 proteins, MCL1, BCL2 and BCL2L1 and that these were expressed in relatively equal levels in TCR activated T cells (Figure 5A). In the absence of ERK signalling the expression of all three of these pro-survival proteins decreased with the most notable drop in BCL2L1 copies (Figure 5A). The data also show that the expression of the pro-apoptotic protein BID and BCL2L11 (BIM) in TCR activated cells was increased in the absence of ERK signalling (Figure 5B). These data are consistent with observations by D'Souza et al. [8], who showed previously that ERK signalling decreased expression of Bcl2 and Bclx mRNA and increased levels of Bim mRNA. The present proteomic data thus provide strong evidence to support the hypothesis that an important role for ERK signalling is to control T cell survival by regulating the expression of the key proteins that mediate this process.

**Figure 6.**
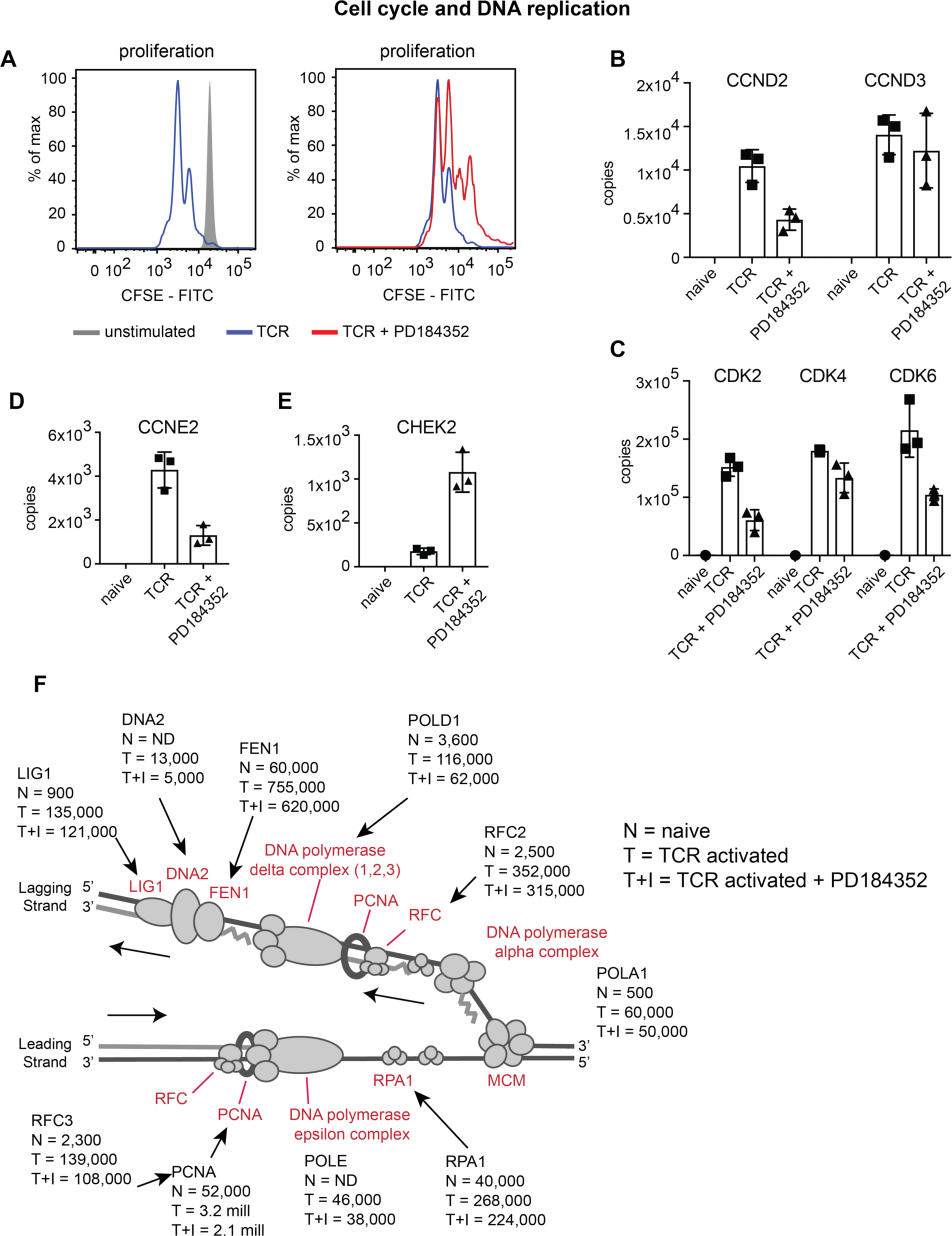
ERK activity has a selective impact on cell cycle and DNA replication machinery. (**a**) Proliferation of CD8^+^ T cells was assessed after 48 h of antigen activation by flow cytometric analysis of CFSE label fluorescence. (**b**,**c**) The impact of ERK activity on key cell cycle proteins. CDK2, 4 and 6, cyclin-dependent kinase 2, 4 and 6; CCND2 and 3, cyclin D2 and cyclin D3. (**d**) The abundance of CCNE2, cyclin E2, and (**e**) CHEK2, checkpoint kinase 2. (**f**) Expression of components of the DNA replication fork complex in naïve (N), TCR activated (T) and TCR activated + inhibitor (T + I) cells. Copy numbers are the mean of 3 biological replicates +/− standard deviation.

To understand the molecular basis for diminished CD8^+^ T cell proliferative expansion (Figure 6A), we assessed the role of ERK signalling in controlling the expression of key T cell cycle regulatory proteins. It is established that ERK control of cyclin D1 expression is important for proliferation in fibroblasts [38]. However, T cells do not express cyclin D1, and rather cyclins D2 and 3 dominate and are key for cell cycle progression in T cells [17]. T cell activation induces increased expression of cyclin D2, cyclin D3 and their associated kinases CDK4 and CDK6 (Figure 6B,C). Blocking ERK1/2 activity resulted in decreased expression of cyclin D2 (CCND2) and cyclin E2 (CCNE2) another G1 cyclin (Figure 6B,D) and reduced expression of DNA replication helicase/nuclease 2 (DNA2) and DNA polymerase delta subunits 1 and 2 (POLD1 and POLD2), critical enzymes in DNA replication and repair (Figure 6F). In addition, blocking ERK activity was found to trigger an increase in the expression of checkpoint kinase 2 (CHEK2), from just a few hundred copies to over 1000 copies (Figure 6E). CHEK2 is a DNA damage response protein and cell cycle checkpoint regulator which when activated can arrest cell cycle progression [39]. These changes afford an explanation for the ERK signalling requirement for optimal T cell proliferative expansion. However, a salient point is that there are clearly ERK independent pathways controlling the expression of critical machinery needed for T cell cycle progression and DNA replication. Hence the expression of cyclin D3 (CCND3) and most components of the DNA replication fork complex were not significantly impacted when ERK activity was blocked (Figure 6B, C and F).

## Discussion

The current study has mapped how ERK1/2 regulate antigen-driven proteome remodelling of CD8^+^ T cells to understand how these evolutionarily conserved kinases control T cell differentiation. The data show that a large proportion of the proteome restructuring that is driven by triggering of the TCR is not dependent on ERK activation. However, the targets for ERK signalling include many of the critical molecules needed for CD8^+^ T cell effector function. The ERKs are thus necessary for the production of proinflammatory cytokines including IL-2 and IFN-γ , and also control the expression of CTL effector molecules including granzymes and perforin. ERK activation was also required for optimal expression of key transcription factors that drive T cell differentiation. For example, IRF8 integrates antigen receptor and cytokine signals to drive CD8^+^ T cell differentiation [29] and its expression is ERK dependent. Similarly, the expression of NFIL3, which controls the expression of perforin in CD8^+^ T cells [30], requires ERK activation. It was also striking that inhibition of ERK1/2 caused activated T cells to increase expression of the transcription factors TCF7/TCF1 and KLF transcription factors which are associated with the commitment of CD8^+^ T cells to a transcriptional program associated with memory T cells [40,41]. However, it should be noted that ERK inhibited CD8^+^ T cells up-regulated expression TOX which is more a marker of exhausted CD8^+^ T cells [42–44]. The combined effects of ERK inhibition on the expression of positive transcriptional regulators of CD8^+^ T cell differentiation and transcription regulators that drive T cell quiescence/exhaustion programs thus affords some explanations for why inhibition of ERKs impacts T cell differentiation [9].

In this context, it was intriguing to observe selectivity in the requirements of ERK signalling for T cell activation. One example of this selectivity is in the context of the expression of critical components of cytokine receptor complexes. ERK signalling pathways thus do not always have equivalent importance for the expression of different cytokine receptor subunits. This selectivity highlights the importance of understanding whether down-regulation of a single subunit of a receptor complex is predictive of loss of receptor function. To answer this question, one needs to have quantitative data that permits an understanding of receptor subunit stoichiometry and then consider what is rate limiting for the production of a high-affinity cytokine receptor complex. For example, naive T cells express IL2Rβ and IL2Rγ subunits and JAK1 but have very low levels of JAK3 and no IL2Rα chain. Hence naive CD8^+^ T cells do not have the molecules needed to form a high-affinity IL-2 receptor. In response to antigen receptor engagement, T cells induce expression of high levels of IL2Rα and also increase the abundance of IL2Rβ, IL2Rγ and the essential tyrosine kinases JAK1 and JAK3. It is however valuable to see from the numbers that the limiting IL-2 receptor subunit for the formation of a high-affinity IL-2 receptors in antigen-activated CD8^+^ T cells will be IL2Rβ which is expressed at approximately 10 000 copies per cell. In comparison, the activated T cells express more than 100 000 copies of IL2Rα, 30 000 copies of IL2Rγ and approximately 20 000 copies of JAK1 and JAK3. In the absence of ERK signalling, IL2Rα chain abundance reduced more than 5-fold to 18 000 copies per cell but importantly there was no ERK requirement for antigen receptor-induced increases in the abundance of IL2Rβ, IL2Rγ, JAK1 or JAK3. Hence, in the ERK inhibited T cells IL2Rα numbers were still in excess of the limiting IL2Rβ subunit allowing the cells to form a high-affinity IL-2 receptor complex. In this respect, it is quite common to use flow cytometry analysis of expression of IL2Rα/CD25 to monitor IL-2 responsiveness of T cells. Flow cytometry uses high levels of signal amplification by fluorophore coupled antibodies to create sensitive assays of relative levels of expression of proteins but does not give information about receptor numbers. A 5-fold drop in IL2Rα expression by flow cytometry would look impressive but the current discussion highlights that it is necessary to understand how many copies of IL2Rα are present to understand the consequences of any reductions. Indeed, another insight from quantitative T cell proteomic data is that immune activation also controls the ability of cells to respond to cytokines by controlling the expression of the JAK family tyrosine kinases which are integral for cytokine receptor function. Thus, of the 4 members of the JAK family, only JAK1 is expressed at relatively high levels in naive T cells, whereas the other family members, JAK2, JAK3 and TYK2 are very low abundance. T cell activation causes increased expression of all JAK family members and, relevant to the role of ERKs in T cells, the induced expression of JAK2 and TYK2 was dependent on ERK activation. JAK2 activity is necessary for T cell differentiation [45] and this ERK requirement for JAK2 expression would also contribute to ERK control of T cell differentiation.

**Figure 7.**
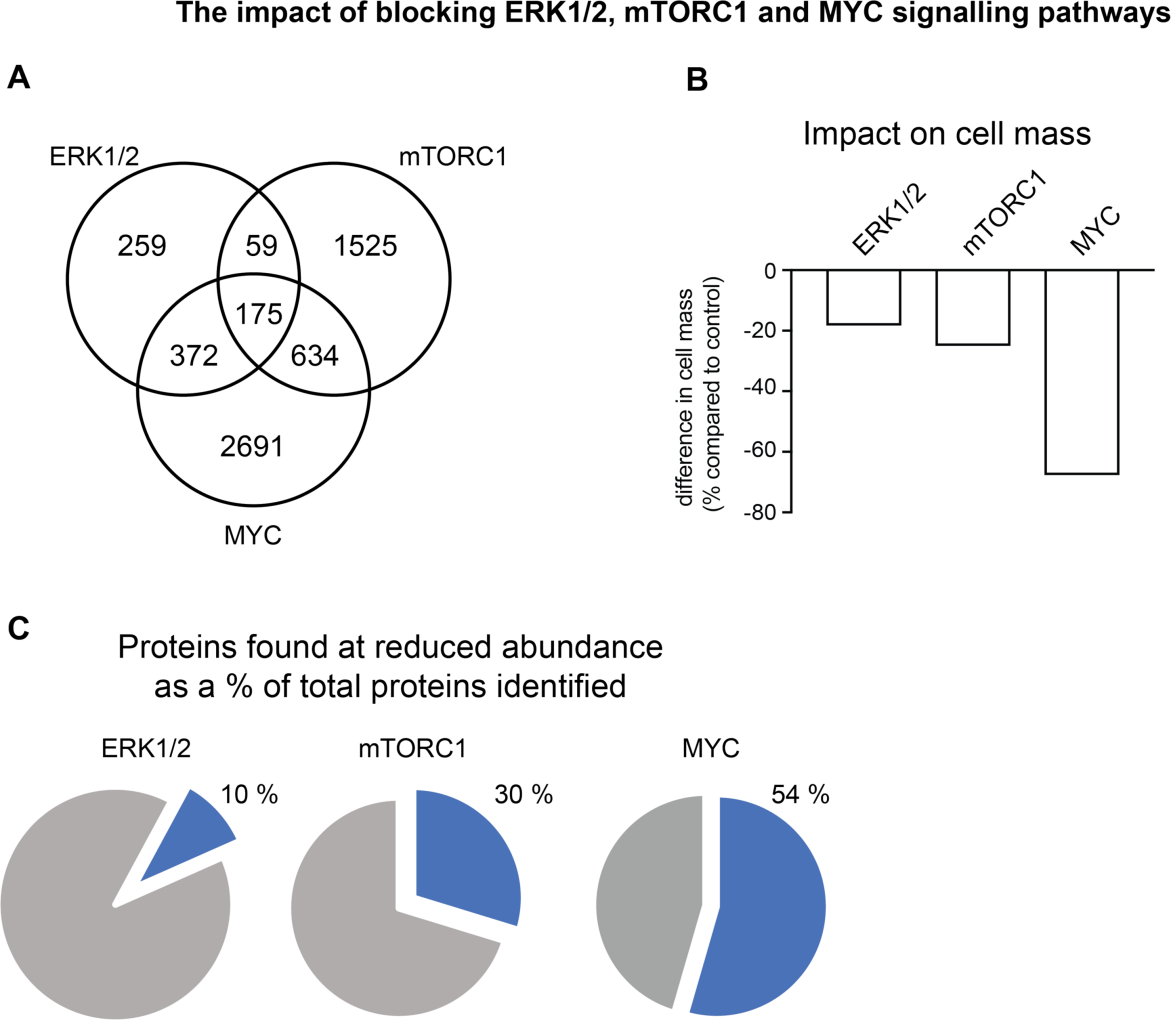
Comparison of the impact of blocking ERK1/2, mTORC1 and MYC signalling pathways on CD8^+^ T cell proteomes. (**a**) Overlap in the number of proteins that are found at the reduced abundance in response to blocking ERK1/2, mTORC1 or MYC signalling pathways. (**b**) The difference in total protein mass of TCR activated CD8^+^ T cells when ERK1/2, mTORC1 or MYC activity is blocked compared with control TCR activated cells. (**c**) The percentage of proteins identified that are found at a reduced abundance when ERK1/2, mTORC1 or MYC activity is blocked compared with control TCR activated cells. For (**a**) and (**c**), proteins were considered to significantly change in abundance with a *P*-value < 0.05 and a fold change <0.66 (2-tailed *t*-test with unequal variance) when compared with the control population, or were exclusively found in control cells at >500 copies per cell and were not detected in cells with either ERK1/2, mTORC1 or MYC pathways blocked.

Finally, a key insight from the present study was that much of the restructuring of the T cell proteome that accompanies T cell activation was ERK1/2 independent. Only 900 of the >8000 proteins quantified in activated T cells were regulated by ERK signalling and many of the major metabolic programs that control cell growth were not dependent on ERK activation. In this respect, we have shown recently that signalling pathways controlled by the transcription factor MYC and the serine/threonine kinase mTORC1 (mammalian target of rapamycin complex 1) are dominant regulators of the metabolic proteome restructuring that accompanies T cell activation [17,19]. In particular, MYC, which controls expression of amino acid transporters in T cells, controlled expression of more than 4000 proteins in TCR triggered CD8^+^ T cells [17,19] and mTORC1 controls expression of 2300 proteins [17]. There were some points of overlap for MYC, mTORC1 and ERK signalling (Figure 7A and Supplementary File S2) but there were also large numbers of unique targets for each pathway. A comparison of how ERKs control T cell proteomes with previous work on how mTORC1 and MYC control T cells [17,19] thus highlights how signal transduction pathways guided by the TCR co-ordinate to control T cell differentiation. However, the distinctiveness of the targets for ERK signalling is that they include some key transcription factors that control T cell differentiation and most strikingly include the cytokines and cytokine receptors that dictate the ability of T cells to communicate externally with other cells of the immune system (Table 5 and Supplementary File S2). The relatively small effect of the ERKs in terms of quantitative effects on T cell mass (Figure 7B,C) merely reflects that the ERK targets are not abundant T cells proteins. They are however key proteins that determine the ability of immune activated T cells to mediate adaptive immune responses.

**Table 5.** Gene ontology enrichment analysis for those proteins that showed reduced abundance when either ERK1/2, mTORC1 or MYC signalling pathways were blocked

Enrichment GO term	Fold enrichment	*P*-value
ERK1/2		
GO:0006955 ∼ immune response	17	6.33 × 10^−10^
GO:0007165 ∼ signal transduction	4	2.2 × 10^−4^
GO:0006959 ∼ humoral immune response	27	3.4 × 10^−4^
GO:0010628 ∼ positive regulation of gene expression	5	3.6 × 10^−4^
GO:0045080 ∼ positive regulation of chemokine biosynth.	67	7.2 × 10^−4^
mTORC1		
GO:0007049 ∼ cell cycle	6	1.7 × 10^−35^
GO:0051301 ∼ cell division	7	5.5 × 10^−35^
GO:0007067 ∼ mitotic nuclear division	8	6.4 × 10^−31^
GO:0007059 ∼ chromosome segregation	10	6 × 10^−14^
GO:0007018 ∼ microtubule-based movement	12	1.2 × 10^−9^
MYC		
GO:0007049 ∼ cell cycle	2	1.2 × 10^−10^
GO:0051301 ∼ cell division	2	6.9 × 10^−8^
GO:0007067 ∼ mitotic nuclear division	2	1.4 × 10^−7^
GO:0006260 ∼ DNA replication	3	2.5 × 10^−5^
GO:0032259 ∼ methylation	3	6.4 × 10^−5^

Proteins that showed the greatest drop in abundance (*P*-value <0.05 and fold change >2 standard deviations from the mean fold change; two-tailed *t*-test with unequal variance) or a presence/absence expression profile, were subject to enrichment analysis for biological processes. The top 5 enriched processes are presented.

## Data availability

All proteomics data is provided in Supplementary File S1. Raw mass spec data files and MaxQuant analysis files are available from the ProteomeXchange data repository (http://proteomecentral.proteomexchange.org/cgi/GetDataset) and can be accessed with the identifier PXD023256 Flow cytometry data are available from the corresponding author upon request.

## Abbreviations

CRABP2: cellular retinoic acid-binding protein 2
CTL: cytotoxic T cells
ERKs: extracellular signal-regulated kinases
HMGCR: hydroxymethylglutaryl-CoA reductase
IL-2: interleukin
KDSR: ketodihydrosphingosine reductase
MAPK: mitogen-activated protein kinases
RA: retinoic acid
TCEP: tris(2-carboxyethyl)phosphine
TCR: T cell antigen receptor
