# Supplementary material for: Extracellular signal-regulated kinase (ERK) pathway control of CD8+ T cell differentiation
Source: Biochem J. 2021 Jan 13;478(1):79–98. doi: 10.1042/BCJ20200661 (PMC7813476; doi:10.1042/BCJ20200661)
Supplement: Supplementary Figures S1-S2 [file BCJ-478-79-s1.pdf]

Supplementary Figure 1

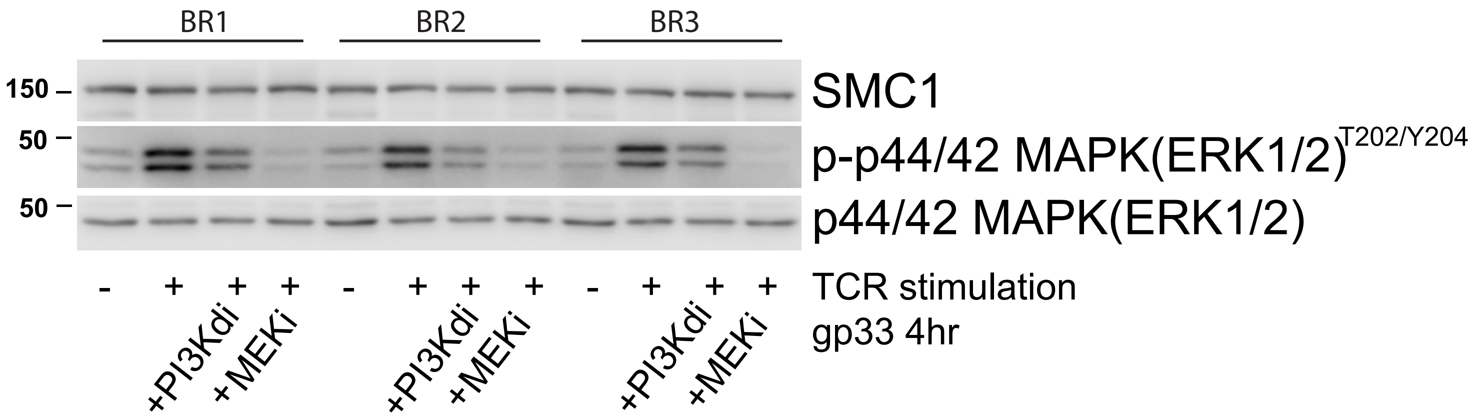

The highly selective MEK inhibitor PD184352 provides effective inhibition of ERK1/2 phosphorylation in CD8+ T cells. Cytotoxic T cells were TCR re-triggered with GP33 peptide +/- PD184352 (+MEKi) or the PI3 kinase delta isoform inhibitor IC87114 (+PI3Kdi) for 4 hours. Cell lysates were analysed by western blot using phopsho-ERK1/2 antibody (p-p44/42 MAPK), pan-ERK antibody (p44/42 MAPK) and SMC1 as a loading control.

Supplementary Figure 2

Naive versus TCR no PD184352

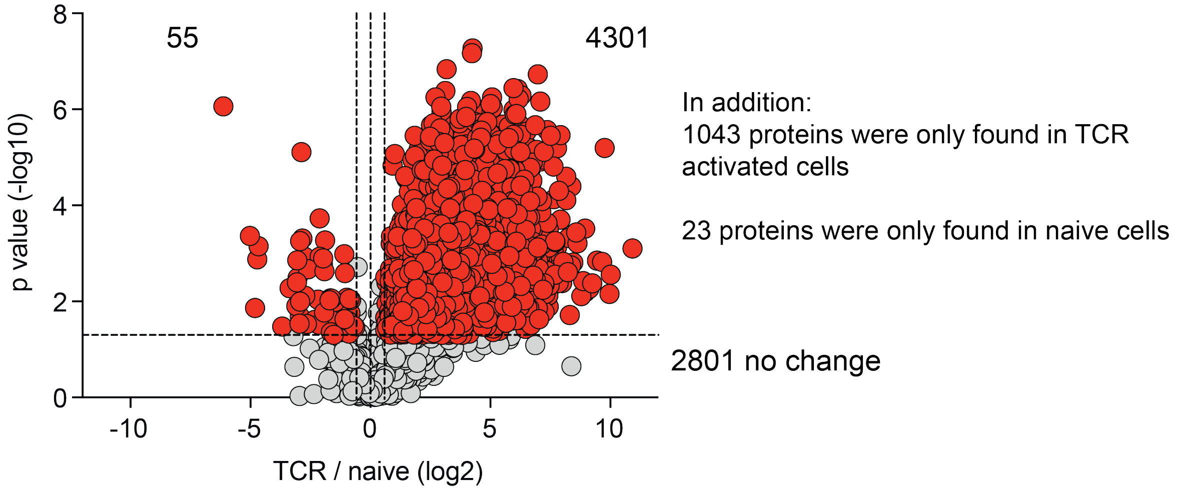

Naive versus TCR + PD184352

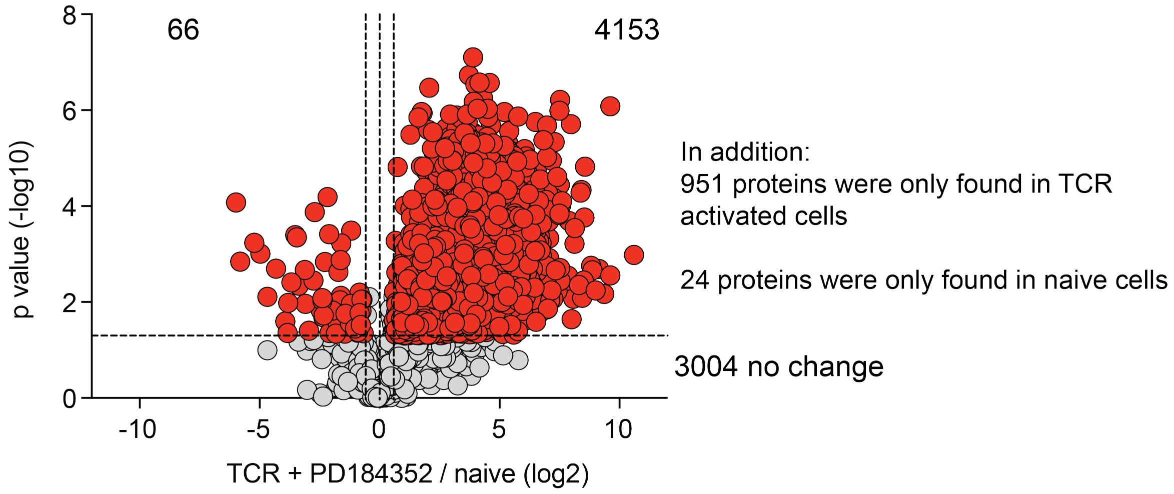

Proteome remodelling of naïve CD8+ T cells in response to antigen activation. Volcano plots show the fold change (log2) and p value (-log10) for naïve versus TCR activated cells +/- the inhibitor PD184352. Proteins were considered to change significantly with a p value <0.05 and a fold change <0.66 or >1.5 (2-tailed t-test with unequal variance). The number of proteins significantly changing is provided along with the number of proteins showing a presence/absence expression profile (>500 copies in each of the 3 replicates of one cell population and not detected in the comparison population).
